# Depression in Chinese patients with type 2 diabetes: associations with hyperglycemia, hypoglycemia, and poor treatment adherence: 中国人2型糖尿病抑郁情况及其与高血糖、低血糖及治疗依从性关系的研究

**DOI:** 10.1111/1753-0407.12238

**Published:** 2015-02-19

**Authors:** Yuying Zhang, Rose ZW Ting, Wenying Yang, Weiping Jia, Wenhui Li, Linong Ji, Xiaohui Guo, Alice PS Kong, Yun‐Kwok Wing, Andrea OY Luk, Norman Sartorius, Donald E Morisky, Brian Oldenburg, Jianping Weng, Juliana CN Chan, China Depression in Chinese Patients with Type 2 Diabetes (DD2) Study Group

**Affiliations:** ^1^ Department of Medicine and TherapeuticsThe Chinese University of Hong Kong, The Prince of Wales HospitalShatinHong Kong SARChina; ^2^ Asia Diabetes FoundationThe Chinese University of Hong Kong, The Prince of Wales HospitalShatinHong Kong SARChina; ^3^ Hong Kong Institute of Diabetes and ObesityThe Chinese University of Hong Kong, The Prince of Wales HospitalShatinHong Kong SARChina; ^4^ Department of Endocrinology and MetabolismChina‐Japan Friendship HospitalBeijingChina; ^5^ Department of Endocrinology and MetabolismShanghai Jiao Tong University Affiliated Sixth People's HospitalShanghaiChina; ^6^ Department of Endocrinology and MetabolismPeking Union Medical College HospitalBeijingChina; ^7^ Department of Endocrinology and MetabolismPeking University People's HospitalBeijingChina; ^8^ Department of Endocrinology and MetabolismPeking University First HospitalBeijingChina; ^9^ Department of PsychiatryThe Chinese University of Hong KongShatin HospitalShatinHong Kong SARChina; ^10^ Association for the Improvement of Mental Health ProgrammesGenevaSwitzerland; ^11^ University of California Los Angeles Fielding School of Public HealthLos AngelesCAUSA; ^12^ School of Population and Global HealthThe University of MelbourneMelbourneVICAustralia; ^13^ Department of Endocrinology and MetabolismThe Third Affiliated Hospital of Sun Yat‐Sen UniversityGuangzhouChina

**Keywords:** depression, hyperglycemia, hypoglycemia, treatment adherence, type 2 diabetes

## Abstract

**Background:**

We hypothesize that depression in type 2 diabetes might be associated with poor glycemic control, in part due to suboptimal self‐care. We tested this hypothesis by examining the associations of depression with clinical and laboratory findings in a multicenter survey of Chinese type 2 diabetic patients.

**Method:**

2538 patients aged 18–75 years attending hospital‐based clinics in four cities in China underwent detailed clinical‐psychological‐behavioral assessment during a 12‐month period between 2011 and 2012. Depression was diagnosed if Patient Health Questionnaire‐9 (PHQ‐9) score ≥10. Diabetes self‐care and medication adherence were assessed using the Summary of Diabetes Self‐care Activities and the 4‐item Morisky medication adherence scale respectively.

**Results:**

In this cross‐sectional study (mean age: 56.4 ± 10.5[SD] years, 53% men), 6.1% (*n* = 155) had depression. After controlling for study sites, patients with depression had higher HbA_1c_ (7.9 ± 2.0 vs. 7.7 ± 2.0%, *P* = 0.008) and were less likely to achieve HbA_1c_ goal of <7.0% (36.2% vs 45.6%, *P* = 0.004) than those without depression. They were more likely to report hypoglycemia and to have fewer days of being adherent to their recommended diet, exercise, foot care and medication. In logistic regression, apart from young age, poor education, long disease duration, tobacco use, high body mass index, use of insulin, depression was independently associated with failure to attain HbA_1c_ target (Odds Ratio [OR] = 1.56, 95%CI:1.05–2.32, *P* = 0.028). The association between depression and glycemic control became non‐significant after inclusion of adherence to diet, exercise and medication (OR = 1.48, 95% CI 0.99–2.21, *P* = 0.058).

**Conclusion:**

Depression in type 2 diabetes was closely associated with hyperglycemia and hypoglycemia, which might be partly mediated through poor treatment adherence.

## 摘要

**背景：:**

2型糖尿病与抑郁关系密切。我们假设2型糖尿病伴发抑郁会导致血糖控制不理想，部分由于自我管理不好所导致。为了证实这一假设，我们在一个2型糖尿病的多中心调查中对抑郁与临床和实验室检查指标的关系进行了研究。

**方法：:**

本研究纳入2538名年龄为18‐75岁的门诊2型糖尿病患者，来自中国4个城市的6家合作医院，入组时间为2011‐2012年。患者接受详细的临床‐心理‐行为评估。使用患者健康量表（Patient Health Questionnaire‐9，PHQ‐9）评估抑郁状况，PHQ‐9≥10分定义为抑郁。使用糖尿病自护行为量表（Summary of Diabetes Self‐care Activities，SDSCA）评估自我照顾情况以及4个项目的Morisky评分评估药物依从性。

**结果：:**

本研究为横断面研究，患者平均年龄是56.4 ± 10.5[SD]岁，53%为男性，其中6.1% (n = 155) 有抑郁。在校正研究中心后，抑郁患者较非抑郁患者的HA1c显著增高 (7.9 ± 2.0 vs. 7.7 ± 2.0%，*P* = 0.008) ，HbA1c达标（<7.0%）率差（36.2% vs 45.6%，*P* = 0.004）。与非抑郁患者相比，抑郁患者低血糖显著多发，饮食、运动、足部护理以及药物依从性差，差别均有统计学意义。根据logistic回归分析，除了年龄小、教育程度差、病程长、吸烟、体重指数更高和使用胰岛素外，抑郁与血糖不达标显著呈正相关（OR = 1.56, 95%CI：1.05‐2.32，*P* = 0.028），但在进一步校正治疗饮食、运动和药物依从性后，该相关性变得不显著（OR = 1.48，95% CI： 0.99‐2.21，*P* = 0.058）。

**结论：:**

2型糖尿病伴发抑郁与高血糖、低血糖发作密切相关，部分可能由于治疗依从性不佳导致。

**Significant findings of the study:** The present study found depression was closely associated with poor glycemic control and hypoglycemia accompanied by poor treatment adherence in Chinese patients with type 2 diabetes.

**What this study adds:** The findings extend the knowledge on the relationship between depression and diabetes in Chinese, which highlights the need for a holistic approach to manage depression which might improve treatment adherence and glycemic control.

## Introduction

The growing burden of type 2 diabetes and associated premature cardiovascular morbidity and mortality is universal and poses major threats to countries in both health and economic terms.^1^ Diabetes complications and treatment‐associated side effects can have negative effects on both physical and mental health.^2^ Furthermore, demands for adjustments and adaptation to lifestyle and self‐care regimens can cause emotional stress, which may impair patients' motivation to adhere to diabetes self‐management.^3^

Type 2 diabetes is associated with increased risk of depression^4^ and their co‐occurrence can further compromise the control of cardiometabolic risk factors with increased risks of complications and functional impairment.^5^, ^6^ Herein, multiple factors including but not limited to disease duration, use of anti‐diabetic drugs, adherence to medications and lifestyle changes might influence glycemic control although their inter‐relationships have not been well defined. Against this background, we hypothesize that depression might impair an individual's motivation and ability to comply with complex self‐care measures and medications, resulting in fluctuation of glycemic control. In a multicenter cross‐sectional study (Depression in Chinese Patients with Type 2 Diabetes, DD2) involving four large cities in China, we tested this hypothesis by examining the associations of depression with glycemic control and their possible mediating factors in type 2 diabetic patients receiving ambulatory care with detailed documentations of clinical, laboratory, emotional and behavioral measures.

## Methods

### Patients and setting

The study enrolled patients with type 2 diabetes from six tertiary hospitals in four major cities in China: Hong Kong (Prince of Wales Hospital), Beijing (Peking Union Hospital, Beijing People's Hospital and China‐Japan Friendship Hospital), Shanghai (Shanghai Sixth Peoples' Hospital) and Guangzhou (The Third Affiliated Hospital of Sun Yat‐Sen University). Between July 2010 and July 2011, Chinese patients with type 2 diabetes aged between 25 and 75 years who underwent comprehensive assessments at these six centers were invited to participate in the study. Referral sources included specialty out‐patient clinics and community out‐patient clinics. Patients were excluded if they had type 1 diabetes (presentation with diabetic ketoacidosis or continuous requirement of insulin within one year of diagnosis), disabling disease or reduced life expectancy (e.g. severe heart failure, severe respiratory disease, or late stages of cancer) or difficulty in communication. All participants gave written informed consent and the study was approved by the local institutional ethics boards.

### Clinical assessment

All participants underwent comprehensive examination based on the procedures set out by the Joint Asia Diabetes Evaluation (JADE) program. The latter is a web‐based portal containing templates for comprehensive assessment, risk engines, personalized report and decision support.^7^, ^8^, ^9^, ^10^ Sociodemographic details, family history of diabetes and mental illness, cardiovascular‐metabolic indices, complications and comorbidities, lifestyle factors, self‐care and use of medications were documented. Hypoglycemia was assessed by asking patients if they had experienced symptoms of hypoglycemia during the past 3 months. Blood pressures (BP) and anthropometric measurements were collected. Diabetic retinopathy was examined by fundoscopy or retinal photography. Peripheral sensory neuropathy was assessed by graduated tuning fork and monofilament. Sensory neuropathy was defined by two of three criteria: abnormal sensation in lower extremities, reduced sensation on monofilament or tuning fork testing. Cardiovascular disease was defined by a history of coronary heart disease, stroke or peripheral vascular disease; the latter defined as non‐traumatic lower extremity amputation or ankle:brachial index (ABI) less than 0.9.

Urine and blood samples were collected after at least 8 h of overnight fasting for plasma glucose, glycated hemoglobin (HbA_1c_), total cholesterol, low density‐lipoprotein‐cholesterol (LDL‐C), high density‐lipoprotein‐cholesterol (HDL‐C), triglyceride, renal function test and random spot urinary albumin‐to‐creatinine ratio (ACR). Hypertension was defined as systolic BP ≥130 mmHg and/or diastolic BP ≥80 mmHg and/or concurrent use of anti‐hypertensive drugs. Dyslipidaemia was defined as LDL‐C ≥2.6 mmol/L, HDL‐C <1.0 mmol/L, triglyceride ≥2.3 mmol/L and/or concurrent use of lipid regulating drugs. Estimated glomerular filtration rate (eGFR) as expressed in mL/min per 1.73 m^2^ was calculated using the abbreviated Modification of Diet in Renal Disease (MDRD) equation: eGFR = 186×[SCR×0.011]^–1.154^×[age]^–0.203^×[0.742 if female], where SCR was serum creatinine in μmol/L.^11^ Chronic kidney disease (CKD) was defined as eGFR <60 mL/min per 1.73 m^2^ while end‐stage renal disease was defined as eGFR <15 mL/min per 1.73m,^2^ requirement of renal transplant or dialysis. Microalbuminuria was defined as urinary ACR ≥2.5–25.0 mg/mmol in men and ≥3.5–25 mg/mmol in women, while macroalbuminuria was defined as urinary ACR ≥25 mg/mmol.

### Mental and behavioral measurement

Participants were requested to complete validated self‐administered questionnaires including the Patient Health Questionnaire‐9 (PHQ‐9),^12^ the Summary of Diabetes Self‐Care Activities (SDSCA),^13^ and the 4‐item Morisky scale for medication adherence.^14^ The PHQ‐9 is a multipurpose instrument for screening, diagnosing, monitoring and measuring the severity of depression.^12^ It consists of nine questions derived from the DSM‐IV diagnostic criteria for major depression. In our previous validation study in Hong Kong Chinese type 2 diabetic patients, the optimal cutoff score to detect depression was 7 with 82.6% sensitivity and 73.7% specificity.^15^ For comparison purpose, we used the widely accepted cutoff point of 10 (88% sensitivity and 88% specificity in the original validation study with most participants being Caucasians) to detect probable depression in our study.^12^, ^16^

The SDSCA is used to assess the frequencies of diabetes self‐care behaviors including diet, exercise, self‐monitoring of blood glucose (SMBG), foot care, medication adherence and smoking in the previous 7 days.^13^ According to the author, the two items on general diet and another two items on specific dietary recommendations: (i) eating five or more servings of fruits and vegetables; and (ii) eating high‐fat foods should be analyzed separately.^13^ In addition, we included the following question to test knowledge on corrective action for hypoglycemia: “how many days in the previous week did you bring candy with you according to recommendation”. In the SCSDA, there are two items on the use of medications. One relates to the use of oral anti‐diabetic drugs, which does not need to be answered by patients treated with lifestyle modification or insulin only. The other relates to insulin use, which does not need to be answered by patients not treated with insulin. However, many patients erroneously answered these items, probably due to oversight of the remarks. Thus we excluded these two items when we calculated the mean score for each item related to self‐care behavior.

The Morisky medication adherence scale consists of four “yes/no” questions, scored 0 and 1, respectively.^14^ The total score ranges from 0 to 4, with “4” indicating high adherence; “2–3”, intermediate adherence and “0–1” low adherence to medication use. The medication adherence score has been shown to have satisfactory internal consistency with concurrent and predictive validity with 81% sensitivity and 44% specificity for detection of non‐adherence to medication.^14^

### Statistical analysis

All data were analyzed using the Statistical Package for Social Science software (SPSS, Version 20.0, IBM). For descriptive analysis, continuous variables were expressed as mean ± standard deviation (SD), mean ± standard error (SE), or median (inter‐quartile range, IQR), and categorical variables were expressed in number (percentage). Between‐group comparisons were analyzed by χ^2^ square test, Student's *t*‐test, analysis of covariates (ANCOVA) or logistic regression with adjustment for study sites as appropriate.

We examined the associations of depression with self‐care behavior, medication adherence and glycemic control using multivariate logistic regression. We constructed three models sequentially to identify independent factors associated with failure to attain HbA_1c_ target of <7.0%. The covariables in Model 1 included probable major depression plus important demographic characteristics regardless of their significance in the univariate analysis. These included study sites, age, gender, disease duration, education level, and current use of tobacco. Model 2 contained clinical characteristics with significant difference (*P* < 0.1) between patients with and without depression, in addition to variables from Model 1. Model 3 included significantly different self‐care behaviors and medication adherence as well as variables from Model 2. We also explored the independent associations of depression with self‐reported hypoglycemia in logistic regression after adjustment for known risk factors for hypoglycemia including age, gender, disease duration, education, regular alcohol use, HbA_1c_, diabetic complications (eGFR, retinopathy, cardiovascular disease, sensory neuropathy), use of insulin and sulphonylurea, and treatment adherence (diet, exercise, and medication adherence score).^17^ A *P*‐value less than 0.05 (two‐sided) was considered significant.

## Results

After excluding 27 patients with incomplete data, 2538 Chinese patients with type 2 diabetes from Hong Kong (*n* = 586), Shanghai (*n* = 463), Guangzhou (*n* = 464) and Beijing (*n* = 1035) were included in the analysis. The mean age of the participants was 56.4 ± 10.5 years, 53% were male (*n* = 1346), and median diabetes duration was 6.0 (IQR: 2.0–10.0) years. The mean PHQ‐9 score was 2.9 ± 3.8 (Table 1). Using the pre‐specified cutoff value of ≥10, 6.1% (*n* = 155) were considered to have depression with 8.9% (*n* = 52) in Hong Kong and 5.3% (*n* = 103) averaged across other China sites. Among those with probable major depression, less than one fifth (16.9%) were taking psychotropic medications.

**Table 1:** Comparison of demographic and clinical characteristics of participants with or without probable major depression (PHQ‐9 score ≥10)

	Total	PHQ‐9 < 10	PHQ‐9 ≥ 10	*P‐*value	Adjusted *P* *
Number	2538	2383 (93.9)	155 (6.1)	–	
PHQ‐9 score	2.9 ± 3.8	2.1 ± 2.4	14.0 ± 4.1	<0.001	<0.001
Age (years)	56.4 ± 10.5	56.5 ± 10.5	55.4 ± 10.7	0.211	0.165
Men	1346 (53.0)	1267 (53.2)	79 (51.0)	0.595	0.250
Duration of diabetes	6.0 (2.0, 10.0)	5.5 (2.0, 10.0)	6.0 (2.0, 11.0)	0.268	0.342
Education				0.370	0.290
<6 years	414 (16.3)	389 (16.3)	25 (16.1)		
6–11 years	775 (30.5)	720 (30.2)	55 (35.5)		
>11 years	1349 (53.2)	1274 (53.4)	75 (48.4)		
Current smoker	416 (16.4)	385 (16.2)	31 (20.1)	0.198	0.332
Not in active employment	1505 (59.3)	1412 (59.4)	93 (60.0)	0.877	0.233
Family history of diabetes	1285 (50.6)	1202 (50.4)	83 (53.5)	0.453	0.594
Family history of mental illness	103 (4.2)	89 (3.8)	14 (9.7)	0.001	0.017
**Metabolic control**					
Body mass index (kg/m^2^)	25.4 ± 3.7	25.4 ± 3.7	25.9 ± 4.3	0.09	0.151
Waist circumference, men (cm)	91.7 ± 9.6	91.5 ± 9.5	93.4 ± 10.2	0.086	0.086
Waist circumference, women (cm)	86.7 ± 10.0	86.6 ± 9.9	88.0 ± 10.9	0.235	0.123
Systolic BP (mmHg)	130 ± 16	130 ± 16	128 ± 18	0.214	0.857
Diastolic BP (mmHg)	78 ± 10	78 ± 10	78 ± 10	0.621	0.714
HbA_1c_ (%)	7.7 ± 2.0	7.7 ± 2.0	7.9 ± 2.0	0.275	0.008
Total Cholesterol (mmol/L)	4.72 ± 1.13	4.72 ± 1.14	4.64 ± 1.08	0.352	0.877
HDL‐C(mmol/L)	1.23 ± 0.33	1.23 ± 0.33	1.25 ± 0.33	0.427	0.954
LDL‐C (mmol/L)	2.69 ± 0.84	2.69 ± 0.83	2.60 ± 0.95	0.197	0.969
Triglyceride (mmol/L)	1.40 (0.99, 2.08)	1.40 (1.00, 2.07)	1.42 (0.97, 2.19)	0.879	0.084
eGFR(mL/min/1.73m^2^)	121 ± 32	121 ± 32	120 ± 36	0.831	0.741
Urinary ACR (mg/mmo/L)	1.14 (0.58, 2.80)	1.14 (0.57, 2.80)	1.17 (0.59, 2.78)	0.896	0.841
Hypertension	1958 (78.5)	1839 (78.5)	119 (78.8)	0.926	0.753
Dyslipidemia	2253 (91.3)	2116 (91.4)	137 (89.0)	0.290	0.366
Self‐reported hypoglycemia in previous 3 months	484 (19.3)	426 (18.1)	58 (37.9)	<0.001	<0.001
**Complications**					
Coronary heart disease	210 (8.3)	189 (7.9)	21 (13.5)	0.015	0.006
Stroke	92 (3.6)	87 (3.7)	5 (3.2)	0.784	0.798
Sensory neuropathy	333 (13.2)	296 (12.5)	37 (24.5)	<0.001	<0.001
Retinopathy	301 (11.9)	284 (12.0)	17 (11.1)	0.743	0.368
Peripheral vascular disease	380 (15.9)	357 (15.8)	23 (16.4)	0.464	0.908
Chronic kidney disease	58 (2.4)	54 (2.4)	4 (2.6)	0.843	0.632
Microalbuminuria	393 (18.7)	369 (18.9)	24 (16.9)	0.565	0.582
Macroalbuminuria	120 (5.7)	109 (5.6)	11 (7.7)	0.283	0.267
End‐stage renal disease	10 (0.4)	8 (0.3)	2 (1.3)	0.088	0.141
**Medications and adherence**					
Lipid regulating drugs	1116 (44.0)	1035 (43.4)	81 (52.3)	0.032	0.036
Statins	1026 (40.4)	953 (40.0)	73 (47.1)	0.082	0.139
BP lowering drugs	836 (32.9)	785 (33.0)	51 (32.9)	0.992	0.985
RAS inhibitors	536 (21.1)	509 (21.4)	27 (17.4)	0.245	0.152
Oral anti‐diabetic drugs	1776 (70.0)	1667 (70.0)	109 (70.3)	0.923	0.466
Sulphonylureas	727 (28.6)	680 (28.5)	47 (30.3)	0.634	0.571
Use of insulin	774 (30.5)	738 (31.0)	36 (23.2)	0.044	0.858
Psychotropic drugs	183 (7.3)	157 (6.7)	26 (16.9)	<0.001	<0.001
Medication adherence score	4.0 (3.0, 4.0)	4.0 (3.0, 4.0)	3.0 (2.0,4.0)	<0.001	<0.001
Medication adherence				<0.001	<0.001
Low (score 0–1)	175 (7.1)	145 (6.3)	30 (20.0)		
Intermediate (score 2–3)	833 (33.9)	771 (33.4)	62 (41.3)		
High (score 4)	1450 (59.0)	1392 (60.3)	58 (38.7)		
**Target achievement**					
HbA_1c_ <7.0%	1092 (45.0)	1037 (45.6)	55 (36.2)	0.023	0.004
BP <130/80 mmHg	943 (37.3)	882 (37.2)	61 (39.6)	0.546	0.579
LDL <2.6 mmol/L	1145 (48.2)	1066 (47.9)	79 (52.7)	0.259	0.944

Data are presented as mean ± SD, median (interquartile range) or number (%).

*Adjusted for research sites.

Definitions of comorbidities and complications:

1. Coronary heart disease: myocardial infarction, unstable angina, percutaneous coronary intervention, coronary bypass operation.

2. Peripheral vascular disease: lower extremity amputation, absent foot pulses with ankle: brachial index <0.9 and/or lower limb revascularization.

3. Diabetes retinopathy: typical retinal changes including vitrectomy, hemorrhages and exudates.

4. Chronic kidney disease: eGFR <60 mL/min per 1.73 m; microalbuminuria: urinary ACR ≥2.5–25 mg/mmol [men] or ≥3.5–25 mg/mmol [women]; macroalbuminuria: urinary ACR >25 mg/mmol.

5. Sensory neuropathy: two of three abnormalities: abnormal sensation in lower limbs, reduced touch sensation to monofilament, reduced vibration sense to graduated tuning fork.

6. Hypertension: systolic BP ≥130 mmHg and/or diastolic BP ≥80 mmHg and/or concurrent use of anti‐hypertensive drugs.

7. Dyslipidemia: LDL‐C ≥2.6 mmol/L, HDL‐C <1.0 mmol/L, triglyceride ≥2.3 mmol/L and/or concurrent use of lipid regulating drugs.

ACR, albumin: creatinine ratio; BP, blood pressure; eGFR, estimated glomerular filtration rate; RAS, renin angiotensin system.

After controlling for study sites, patients with depression had higher HbA_1c_ (7.9 ± 2.0 vs. 7.7 ± 2.0%, *P* = 0.008) and were less likely to achieve HbA_1c_ goal of <7.0% (36.2% vs 45.6%, *P* = 0.004). Patients with depression were more likely to report hypoglycemia during the previous 3 months than those without depression (37.9% vs 18.1%, *P* < 0.001), despite comparable use of sulphonylurea and insulin. Depression was associated with 1.7–2.0‐fold higher prevalence of coronary heart disease and sensory neuropathy despite similar blood pressures, body mass index, blood lipids and renal function between those with or without depression. Fewer subjects with depression reported high medication adherence score (which indicated high medication adherence) than the non‐depressed group (38.7% vs 60.3%, *P* < 0.001) (Table 1). This was accompanied by fewer days per week of frequent exercises, adherence to recommended diet regimen and foot care, based on the SDSCA questionnaire in the group with depression (Table 2).

**Table 2:** Mean number of days in the prior week when patients adhered to diabetes self‐care behaviors stratified by depression*

	PHQ‐9 < 10	PHQ‐9 ≥ 10	*P*‐value
General diet	4.41 ± 0.05	3.97 ± 0.20	0.033
Fruit and vegetables	3.84 ± 0.06	3.51 ± 0.21	0.133
High‐fat food consumption	2.41 ± 0.05	2.16 ± 0.19	0.211
SMBG	2.23 ± 0.05	2.32 ± 0.18	0.659
Exercise	3.90 ± 0.06	3.31 ± 0.20	0.005
Foot care	3.97 ± 0.05	3.57 ± 0.17	0.022
Bring along candy	2.60 ± 0.07	2.80 ± 0.25	0.239

*Data are presented as mean ± SE adjusted for study sites, age, gender and disease duration.

SMBG, Self‐monitoring of blood glucose.

Stepwise logistic regression showed that depression was an independent factor for failure to attain HbA_1c_ target to <7.0%, in addition to young age, long disease duration, low education level, smoking, higher body mass index, and use of insulin (Table 3, Model 1 and 2). However, the effect of depression became non‐significant after adding self‐care behavior (diet, exercise) and medication adherence to the model (Table 3, Model 3). After adjustment for sites, age, gender, disease duration, education, history of retinopathy, sensory neuropathy, and cardiovascular disease, HbA_1c_, eGFR, medication adherence score, adherence to diet, exercise, use of insulin and sulphonylurea, depression remained independently associated with hypoglycemia (Table 4).

**Table 3:** Stepwise logistic regression for failure to attain HbA1c target(<7.0%)* in Chinese patients with type 2 diabetes (*n* = 2272)

	Model I		Model II		Model III	
	OR(95%CI)	*P*	OR(95%CI)	*P*	OR(95%CI)	*P*
PHQ‐9 score ≥10	1.56 (1.06–2.30)	0.023	1.56 (1.05–2.32)	0.028	1.48 (0.99–2.21)	0.058
**Demographic characteristics**						
Age, years	0.96 (0.95–0.97)	<0.001	0.96 (0.95–0.97)	<0.001	0.96 (0.95–0.97)	<0.001
Men	0.87 (0.71–1.06)	0.165	0.85 (0.69–1.05)	0.123	0.84 (0.68–1.04)	0.104
Duration of diabetes, years	1.09 (1.08–1.11)	<0.001	1.09 (1.07–1.11)	<0.001	1.09 (1.07–1.11)	<0.001
Current smoker	1.65 (1.26–2.15)	<0.001	1.59 (1.21–2.09)	0.001	1.53 (1.16–2.02)	0.003
Low education (<6 years)	1.75 (1.33–2.31)	<0.001	1.64 (1.24–2.18)	0.001	1.58 (1.19–2.11)	0.002
**Clinical characteristics** ^†^						
Body mass index, kg/m^2^			1.10 (1.07–1.13)	<0.001	1.10 (1.07–1.13)	<0.001
Coronary heart disease			1.17 (0.83–1.66)	0.370	1.16 (0.82–1.64)	0.411
Sensory neuropathy			1.22 (0.92–1.63)	0.169	1.21 (0.91–1.61)	0.196
Use of insulin			2.72 (2.17–3.41)	<0.001	2.79 (2.22–3.50)	<0.001
**Self‐care and medication adherence**						
SDSCA general diet					0.95 (0.91–0.99)	0.007
SDSCA exercise					1.01 (0.97–1.05)	0.679
Medication adherence score^‡^					0.90 (0.82–1.00)	0.043

PHQ‐9, Patient Health Questionnaire‐9; SDSCA, Summary of Diabetes Self‐care Activities, score ranges from 0 to 7, with higher score indicating better adherence to recommended self‐care regimen.

*HbA1c ≥7.0% was used as the reference dependent outcome. Clinical characteristics and self‐care behaviors significant at *P* < 0.1 level were sequentially entered into the model. All models were adjusted for study sites.

^†^Waist circumference was not adjusted for due to collinearity with body mass index.

^‡^Medication adherence score (range: 0–4) was used as a continuous variable in the model. Higher score indicates better medication adherence.

**Table 4:** Logistic regression for self‐reported hypoglycemia in Chinese patients with type 2 diabetes (*n* = 2159)*

	OR (95%CI)	*P*‐value
PHQ‐9 ≥ 10	2.62 (1.74–3.96)	<0.001
Age (years)	0.98 (0.97–1.00)	0.017
Men	1.10 (0.85–1.43)	0.474
Disease duration (years)	1.04 (1.02–1.07)	<0.001
Low education (<6 years)	0.79 (0.53–1.17)	0.238
Regular alcohol use	1.28 (0.83–1.96)	0.267
History of diabetic retinopathy	0.89 (0.61–1.29)	0.529
History of sensory neuropathy	1.35 (0.96–1.88)	0.084
History of cardiovascular disease	1.16 (0.85–1.60)	0.351
HbA_1c_ (%)	0.85 (0.78–0.93)	<0.001
eGFR (mL/min per 1.73 m^2^)	1.00 (1.00–1.01)	0.711
Use of insulin	2.28 (1.69–3.08)	<0.001
Use of sulphonylurea	1.72 (1.31–2.24)	<0.001
SDSCA general diet	1.00 (0.95–1.05)	0.911
SDSCA exercise	1.00 (0.95–1.05)	0.966
Medication adherence score^†^	1.01 (0.90–1.15)	0.815

*The model was adjusted for study sites. Any self‐reported hypoglycemia during the previous 3 months was used as the reference.

^†^Medication adherence score (range: 0–4) was used as a continue variable in the model. Higher score indicates better medication adherence.

eGFR, estimated glomerular filtration rate; SDSCA, Summary of Diabetes Self‐care Activities. Score ranges from 0 to 7, with higher score indicating better adherence to recommended self‐care regimen.

## Discussion

In this study comprising more than 2500 patients with type 2 diabetes recruited from six hospitals in four metropolitan cities in China, over 6% had depression based on a validated questionnaire, but less than one fifth of these patients were treated with psychotropic medications. Patients with high PHQ‐9 score were more likely to have poor glycemic control and hypoglycemia, which were associated with poor self‐care and medication non‐adherence. Given the possibility of under diagnosis of this silent condition, together with the high prevalence of diabetes in China^18^ and the additive effects of these two comorbid conditions on mortality and morbidity,^19^ these data have important public health implications.

### Prevalence of depression in type 2 diabetes

In a multicentre Translating Research Into Action for Diabetes (TRIAD) study including over 8700 adults with diabetes, predominantly Caucasians recruited from different managed care settings in US, 18% had major depression based on a cutoff score of 10 using the PHQ‐8.^20^ In a previous analysis involving a subset of patients in Hong Kong, we have validated the PHQ‐9 in diagnosing depression using interview by psychiatrists as gold reference with a lower cutoff point of ≥7.^15^ In this multicenter analysis, to allow for comparisons with literature, we used a cutoff score of ≥10, which identified 6.1% of subjects as having probable major depression. However, if we adopt a cut‐off value of seven which fits Chinese samples,^15^ the prevalence of probable depression increased to 13.0% (18.1% in Hong Kong and 11.5% in other China sites), which was higher than that in the general population^21^ and was quite close to data reports from other countries.^20^

Some researchers suggested that the lower prevalence of depression in Chinese compared to the Western population might be in part due to cultural differences in the depressive symptom expression.^22^, ^23^, ^24^ For instance, social stigma associated with depression and the tendency to somatize depressive symptoms may contribute to its under‐diagnosis in Chinese population.^23^, ^25^ Traditional Chinese culture encourages withstanding of hardship, patience and forbearance as well as maintaining social and family harmony through interdependence. These cultural values may contribute to tolerance for emotional burden and/or under‐reporting of depression.^23^ In support of this notion, we found that three items of the PHQ‐9 (sleep, energy, appetite) contributed significantly to the diagnosis of depression in Hong Kong Chinese in our previous analysis,^15^ suggesting that more sensitive tools or lower cut off values will need to be used in Chinese subjects. Besides, other methodological issues such as subject characteristics, settings and disease contexts will need to be taken into consideration when interpreting these results.

In this study, patients residing in Hong Kong had a higher rate of depression than those recruited in Mainland China and we speculated that this might be related to the less favorable socio‐economic status, lower education level and higher proportions of housewife and unemployment in the Hong Kong sub‐cohort.^24^ Besides, Hong Kong has been a British colony for over a century and its culture and social values are invariably westernized. More patients from Hong Kong reported stresses from work, home and finance and they were more likely to have family history of mental illnesses (supplementary Table S1).

### Depression and glycemic control

In this study population, depression was associated with poor glycemic control but without differences in other metabolic indices, such as blood pressure and lipid. These results are consistent with reports by others.^26^, ^27^ In an earlier meta‐analysis of 24 cross‐sectional studies involving 2817 patients, Lustman and colleagues found that depression was significantly correlated with poor glycemic control in both type 1 and type 2 diabetes,^26^ although most of the included studies had small sample sizes. In the larger scale TRIAD study involving over 8700 subjects, patients with high PHQ‐8 scores also had worse glycemic control than those with low score.^20^ In a prospective cohort of over 3700 type 2 diabetic patients recruited from primary care settings, patients identified to have minor or major depression at baseline had higher HbA_1c_ during a 5‐year follow‐up period, although this was rendered non‐significant after correction for disease duration, history of cardiovascular disease, hypertension and anti‐diabetic treatment.^27^

### Depression and adherence

Other researchers have suggested that suboptimal glycemic control in co‐morbid depression might be partly explained by non‐adherence with self‐care and medications.^28^ In a meta‐analysis of 47 cohort studies, depression was associated with poor self‐care, medication non‐adherence and frequent defaults.^29^ Congruent to these observations, we also found that the group with probable depression were less likely to adhere to general diet and physical exercise, and had higher rates of medication non‐adherence. The fact that the association of depression with failure to attain HbA_1c_ target was rendered non‐significant after adjustment for SDSCA diet and exercise subscale score as well as medication adherence score further suggested that poor glycemic control in patients with depression might be mediated by suboptimal adherence to self‐care and medication. Because of this, the complex anti‐diabetic regimen and polypharmacy, often applied to patients with a long disease duration and with multiple risk factors, can be associated with unwanted side‐effects including hypoglycemia and weight gain.^17^, ^30^ In our cohort, one‐fifth of patients with probable depression were treated with insulin, which might be associated with social stigma and met with resistance due to pain, inconvenience and interference with daily life.^31^

### Hypoglycemia and depression

Apart from high HbA_1c_, patients with probable depression were twice more likely to report hypoglycemia than the non‐depressed group, even after adjustment for potential confounders, including disease duration, renal function, use of insulin and sulphonylurea. The independent association of depression on symptomatic and severe hypoglycemia had been well reported. In a prospective study of 4117 adults with diabetes, depression was associated with a hazard ratio of 1.42 for hospitalization for hypoglycemia.^32^ In the Study to Help Improve Early evaluation and management of risk factors Leading to Diabetes (SHIELD) survey of 2718 patients with type 2 diabetes, those reporting hypoglycemia during the previous 1 year had higher PHQ‐9 scores than those without hypoglycemia.^33^

In most clinical trials conducted in controlled settings, hypoglycemia was often associated with tight glycemic control.^34^ In our study, we also found this association although we also noted high glycemic variability in patients with depression, which might be due to multiple reasons, such as suboptimal treatment adherence with sporadic intake of medications, erratic meal plan, insufficient preventative measures for hypoglycemia and poor motivation to improve self‐management.^35^ Besides, fear of hypoglycemia might also lead to unwillingness to intensify treatment or over‐correction for hypoglycemia. From a biological standpoint, high cortisol levels and dysregulation of the autonomic nervous system might also lead to fluctuations in glycemic control.^36^ In support of this notion, both hypoglycemia and glycemic variability were independent predictors for mortality and cardiovascular‐renal events in Chinese type 2 diabetic patients.^37^, ^38^

### Study limitations

Several limitations of this study need to be acknowledged. First, all our patients were recruited from hospital‐based clinics and thus might not be representative of the general diabetic population in China. That said, in China including Hong Kong, primary health care for managing chronic conditions like diabetes still remains relatively under‐developed and many diabetic patients still receive their care in hospital‐based clinics, irrespective of disease severity. On the other hand, we only involved clinics from metropolitan cities and thus patients living in rural areas were not represented. Second, our JADE database did not have data on marital status and income, which might have prognostic significance for depression.^39^ Third, patients with probable depression did not receive formal psychiatric examination to confirm a diagnosis of major depression. However we have chosen to take a high score of the PHQ‐9(≥10) to indicate depression, which is higher than the optimal cutoff score (≥7) previously validated in the same group of Hong Kong patients. Lastly, due to the cross‐sectional nature of our study, we were not able to establish any definitive causal relationships.

## Conclusions

In this observational study, depression was associated with both hyperglycemia and hypoglycemia accompanied by suboptimal self‐care and treatment adherence. In diabetic patients with variable glycemic control, physicians should consider the possibility of underlying depression and adopt a more holistic approach to manage depression and emotional distress which might improve both medication adherence and self‐care. The fact that less than one fifth of patients with depression were receiving treatment for it underlines the importance of this recommendation.

## Disclosure

J.C. is the Chief Executive Officer of the Asia Diabetes Foundation on a pro bono basis. She has received honorarium for consultancy or giving lectures from Bayer, Boehringer Ingelheim, Daiichi‐Sankyo, Eli‐Lilly, GlaxoSmithKline, Merck Sharp & Dohme, Merck Serono, Pfizer, Astra Zeneca, Sanofi, Novo‐nordisk and/or Bristol‐Myers Squibb. A.K. has received honorarium for consultancy or giving lectures from Nestle Nutrition Institute, Merck Serono, Pfizer, Eli Lilly, Roche, Sanofi, Janssen and AstraZeneca. Other authors declare that they have no conflict of interest.

## Supporting information

Appendix Table S1. Comparison of socio‐demographic and clinical characteristics of participants in Mainland China and Hong Kong.

## Appendix

### Members of DD2 study group

Juliana Chan, Wenying Yang, Weiping Jia, Wenhui Li, Linong Ji, Xiaohui Guo, Jianping Weng, Xiaoping Xing, Yun‐Kwok Wing, Norman Sartorius, Andrea Luk, Yuying Zhang, Rose Ting, Ronald Ma, Wing‐yee So, Alice Kong, Risa Ozaki, Hairong Nan, Marco Lam, Joyce Lam, Mandy Yu, Yu Zhu, Mingzi Li, Xuhong Hou, Jing Hong, Junqing Zhang, Yanhua Zhu.
