# Supplementary material for: Depression in Chinese patients with type 2 diabetes: associations with hyperglycemia, hypoglycemia, and poor treatment adherence: 中国人2型糖尿病抑郁情况及其与高血糖、低血糖及治疗依从性关系的研究
Source: J Diabetes. 2015 Feb 19;7(6):800–8. doi: 10.1111/1753-0407.12238 (PMC4964948; doi:10.1111/1753-0407.12238)
Supplement: Supplementary file 1 — Appendix Table S1. Comparison of socio‐demographic and clinical characteristics of participants in Mainland China and Hong Kong. [file JDB-7-800-s001.docx]

**Supplementary**

**Table S1. Comparison of socio-demographic and clinical characteristics of participants in Mainland China and Hong Kong.**

|  | | **Mainland**  **(n=1952)** | **Hong Kong**  **(n=586)** | ***P* value** |
| --- | --- | --- | --- | --- |
| Age (years) | | 56.8±10.8 | 55.0±9.5 | <0.001 |
| Men | | 999 (51.2) | 347 (59.2) | 0.001 |
| Duration of diabetes (years) | | 5.0 (1.0,10.0) | 7.0 (3.0, 11.0) | <0.001 |
| Education | |  |  | <0.001 |
| <6 years | | 255 (13.1) | 159 (27.1) |  |
| 6-11 years | | 487 (25.0) | 289 (49.3) |  |
| >11 years | | 1209 (62.0) | 138 (23.5) |  |
| Current smoker | | 354 (18.1) | 62 (10.6) | <0.001 |
| Occupation | |  |  | <0.001 |
| Employed (full time or part time) | | 1236 (63.4) | 270 (46.1) |  |
| Housewife | | 101 (5.2) | 119 (20.3) |  |
| Retired | | 1107 (56.8) | 132 (22.5) |  |
| Unemployed | | 29 (1.5) | 19 (3.2) |  |
| Family history of mental illness | | 62 (3.3) | 41 (7.1) | <0.001 |
| **Metabolic control** | |  |  |  |
| Body mass index (kg/m^2^) | | 25.2±3.5 | 26.0±4.4 | <0.001 |
| Systolic BP (mmHg) | | 129±17 | 131±16 | 0.042 |
| Diastolic BP (mmHg) | | 77±9 | 80±10 | <0.001 |
| HbA1c (%) | | 7.7±2.1 | 7.5±1.4 | <0.001 |
| Total Cholesterol (mmol/L) | | 4.79±1.20 | 4.51±0.89 | <0.001 |
| HDL-C (mmol/L) | | 1.19±0.31 | 1.34±0.37 | <0.001 |
| LDL-C (mmol/L) | | 2.75±0.86 | 2.49±0.72 | <0.001 |
| Triglyceride (mmol/L) | | 1.45 (1.01, 2.19) | 1.20 (0.90, 1.80) | <0.001 |
| eGFR (ml/min/1.73m^2^) | | 125±34 | 108±24 | <0.001 |
| Urinary ACR (mg/mmo/L) | | 1.14 (0.62, 2.57) | 1.10 (0.49, 3.90) | <0.001 |
| Hypertension | | 1492 (78.2) | 466 (79.5) | 0.495 |
| Dyslipidaemia | | 1695 (92.3) | 496 (84.9) | <0.001 |
| Self-reported hypoglycemia in previous 3 months | | 364 (19.0) | 120 (20.5) | 0.424 |
| **Complications** | |  |  |  |
| Coronary heart disease | | 177 (9.1) | 33 (5.6) | 0.008 |
| Stroke | | 72 (3.7) | 20 (3.4) | 0.754 |
| Sensory neuropathy | | 317 (16.3) | 16 (2.7) | <0.001 |
| Peripheral vascular disease | | 228 (11.7) | 8 (1.4) | <0.001 |
| Chronic kidney disease | | 45 (2.4) | 13 (2.2) | 0.761 |
| Microalbuminuria | | 267 (17.6) | 126 (21.5) | 0.042 |
| Macroalbuminuria | | 69 (4.6) | 51 (8.7) | <0.001 |
| **Medications and adherence** | | |  |  |
| Statins | 821 (42.1) | | 205 (35.0) | 0.002 |
| BP lowering drugs | 581 (29.8) | | 255 (43.5) | <0.001 |
| RAS inhibitors | 337 (17.3) | | 199 (34.0) | <0.001 |
| Oral antidiabetic drugs | 1400 (71.7) | | 376 (64.2) | <0.001 |
| Use of insulin | 690 (35.3) | | 84 (14.3) | <0.001 |
| Psychotropic drugs | 135 (7.0) | | 48 (8.3) | 0.285 |
| Psychotropic drugs among patients with PHQ-9 score≥10 | 15 (14.7) | | 11 (21.2) | 0.312 |
| **Target achievement** |  | |  |  |
| HbA_1c_<7.0% | 856 (46.6) | | 236 (40.3) | 0.008 |
| BP<130/80 mmHg | 720 (37.1) | | 223 (38.1) | 0.680 |
| LDL<2.6 mmol/L | 829 (45.9) | | 316 (55.7) | <0.001 |
| **External stress** |  | |  |  |
| Home stress (moderate or above) | 126 (6.5) | | 67 (11.4) | <0.001 |
| Work stress (moderate or above) | 184 (9.7) | | 113 (19.7) | <0.001 |
| Financial stress (moderate or above) | 375 (19.4) | | 250 (43.1) | <0.001 |

Data are presented as mean±SD, median (interquartile range) or number (%).

BP, blood pressure; RAS, renin angiotensin system; eGFR, estimated glomerular filtration rate; ACR, albumin: creatinine ratio.

Definitions of comorbidities and complications:

1. Coronary heart disease: myocardial infarction, unstable angina, percutaneous coronary intervention, coronary bypass operation.
2. Peripheral vascular disease: lower extremity amputation, absent foot pulses with ankle: brachial ratio<0.9 and/or lower limb revascularization.
3. Sensory neuropathy: 2 of 3 abnormalities: abnormal sensation in lower limbs, reduced touch sensation to monofilament, reduced vibration sense to graduated tuning fork.
4. Chronic kidney disease: eGFR<60 ml/min/1.73 m^2^; microalbuminuria: urinary ACR ≥2.5-25 mg/mmol [men] or ≥3.5-25 mg/mmol [women]); macroalbuminuria: urinary ACR >25 mg/mmol.
5. Hypertension: systolic BP≥130 mmHg and/or diastolic BP≥80 mmHg and/or concurrent use of anti-hypertensive drugs.
6. Dyslipidaemia: LDL-C≥2.6 mmol/L, HDL-C<1.0 mmol/L, triglyceride≥2.3 mmol/L and/or concurrent use of lipid regulating drugs.
